# Prediction of Communicative Disorders Linked to Autistic Spectrum Disorder Based on Early Psychomotor Analysis

**DOI:** 10.3390/children9030397

**Published:** 2022-03-11

**Authors:** Darío Rincón-Rufo, Violeta Vera-Pérez, Alicia Cuesta-Gómez, María Carratalá-Tejada

**Affiliations:** 1 José de Echegaray Public School, 28051 Madrid, Spain; dariorin91@gmail.com; 2 Ciudades Unidas Public School, 28830 Madrid, Spain; miss.violet.vera@gmail.com; 3 Motion Analysis, Ergonomics, Biomechanics and Motor Control Laboratory (LAMBECOM), Department of Physical Therapy, Occupational Therapy, Rehabilitation and Physical Medicine, Faculty of Health Sciences, Rey Juan Carlos University, 28922 Alcorcón, Spain; maria.carratala@urjc.es

**Keywords:** autism spectrum disorder, psychomotor skills, language disorders, prediction, children

## Abstract

This systematic review evaluated psychomotor differences between children with and without siblings who have autism spectrum disorder (ASD), as well as the most reliable psychomotor skills that can help predict ASD and its associated language disorders. Literature from 2005 to 2020 was searched using the following databases: PubMed, Trip Medical Database, Cochrane, Web of Science, Science Direct, and Brain. A total 11 papers were included. Fine motor skills and joint attention displayed reliable results in order to predict ASD and its associated language disorders. The period between the first and the second year of life was considered the most appropriate one for the assessment of psychomotor skills. The best period to predict language disorders and ASD diagnosis is around 36 months old.

## 1. Introduction

Autism spectrum disorder (ASD) is a neurodevelopment disorder characterized by qualitative difficulties in speech and social interaction areas, as well as restricted and repetitive interest ranges [1].

Quite possibly, the first reference to this disorder goes back to the 16th century, when Johannes Mathesius (1504–1565) wrote the story of a twelve-year-old child with severe symptoms that resembled autistic features [2]. In 1934, Leo Kanner would, for the first time, define children with this disorder as “children [who] have come into the world with an innate inability to form the usual, biologically provided contact with people” [3,4].

Nowadays, the International Classification of Functioning, Disability, and Health (ICF), developed by the World Health Organization (WHO), defines the ASD disorder in their latest version of their International Classification of Diseases (ICD-11) as a disorder “characterized by persistent deficits in the ability to initiate and to sustain reciprocal social interaction and social communication, and by a range of restricted, repetitive, and inflexible patterns of behavior, interests or activities that are clearly atypical or excessive for the individual’s age and sociocultural context” [5].

Meanwhile, the American Psychological Association (APA) classifies ASD and its diagnosis criteria in the fifth edition of their Diagnostic and Statistical Manual (DSM-V). In this new update, the previous diagnosis for childhood disintegrative disorder, pervasive developmental disorder—not otherwise specified (PDD—NOS), Asperger syndrome, and autistic disorder fall under the term ASD [6]. Regarding its diagnosis, the DSM-V establishes the following criteria: persistent deficits in social communication and social interaction; restricted, repetitive patterns of behavior, interests, or activities; symptoms must be present in the early developmental period; symptoms cause clinically significant impairment in social, occupational, or other important areas of current functioning; these disturbances are not better explained by intellectual disability (intellectual developmental disorder) or global developmental delay [7].

Regarding the etiology of the disorder, recent studies are gravitating towards two potential causes: prenatal and environmental factors, and epigenetic and genetic factors [8]. These last two appear to be of special relevance [8,9,10], which in turn means that children with direct relatives with ASD are at a high risk (HR) of presenting the disorder [11,12,13]. Nonetheless, the neurobiology of this disorder is far from being completely known [8].

Subjects with ASD are mostly characterized as showing difficulties related to social communication and language; as well as other related medical and psychiatric issues, such as anxiety, depression, attention deficit hyperactivity disorder, and gastrointestinal or sleeping problems [14,15,16]. Regarding the communicative aspect, they frequently show deficits in both receptive and expressive language, although some subjects can present test scores within average ranges [17].

As for motor skills, ASD is linked to certain deficits in gross and fine motor skills, coordination, postural control, and static balance [18]. In this field, the development of motor skills is considered an essential element for the achievement of communicative and social factors [19,20]. In order to adequately situate this systematic review, it is necessary to talk about those investigations that relate the development of gross motor skills [19], gait and stable sitting [21], and gross and fine motor skills [22] with language development.

The Autism Diagnostic Observation Schedule (ADOS-2), and the Autism Diagnostic Interview-Revised (ADI-R) [23] are among the most widespread tools for diagnosis. The ADOS-2 is a standardized observational tool divided into five modules, which are adapted to the age and/or language development level of the subject at the time of the test. The ADI-R tool is designed to detect ASD through personal interviews with family members or carers of a subject from 2 years of age [24].

Based on the risk that genetic inheritance represents in children who have close relatives with ASD, this systematic review suggests a possible relationship between the development of early psychomotor skills during the first three years of life, and the potential children with brothers and/or sisters with ASD have of having language disorders and/or ASD. This proposal is based on the high risk of having ASD that these children present due to their etiological genetic factors.

This initial goal leads to the exposition of a PICO question to guide the intervention, which should in turn reveal whether an early analysis of the psychomotor skills in children at a high risk of having ASD can predict language disorders related to the disorder. To do so, motor, linguistic, and ASD diagnosis assessment scales will be used.

The goal of this systematic review is to determine whether there are differences during the first three years of psychomotor development in children at a high risk of having ASD, which could guide the prediction of potential speech deficits and the later diagnosis of ASD.

To achieve this goal, a systematic review will be carried out, taking into account not only the results extracted from the selected bibliography, but their methodological quality and their risk of bias.

## 2. Materials and Methods

### 2.1. Search Strategy

A systematic literature search was carried out on February 2020, focusing on articles published between January 2005 and February 2020 that might answer the PICO question aforementioned. The search strategy included both Spanish and English literature from PubMed, Trip Medical Database, Cochrane, Web of Science, Science Direct, and Brain. Specific terms were used. Those terms were determined before starting the literature search, based on the definitions DSM-V gives for ASD, the goal of this systematic review, and the MeSH criteria. The selected terms were: (1) “autism spectrum disorder” AND “motor skills” AND “language disorder”; (2) “autistic disorder” AND “motor skills” AND “language disorder”; (3) “Asperger syndrome” AND “motor skills” AND “language disorder”; (4) “pervasive development disorder” AND “motor skills” AND “language disorder”; (5) “autism spectrum disorder” AND “psychomotor disorder” AND “language disorder”; (6) “autistic disorder” AND “psychomotor disorder” AND “language disorder”; (7) “Asperger syndrome” AND “psychomotor disorder” AND “language disorder”; (8) “pervasive development disorder” AND “psychomotor disorder” AND “language disorder”.

Besides reviewing the articles on the selected literature, a secondary review of these articles’ literature was carried out in order to assess their potential integration in the systematic review.

### 2.2. Eligibility

Studies were eligible if they were longitudinal observational analytical studies. Those studies assessed psychomotor performance in children for the development of a subsequent analysis of communicative skills, establishing a relationship between both data. Selected studies should evaluate any psychomotor skill and its relation with the child’s communicative skills up to 36 months of age. Specific language disorders diagnosis tests and ASD diagnosis tests were accepted for the evaluation of communicative skills. Every chosen article should select subjects at high risk of ASD due to having siblings with a positive diagnosis.

### 2.3. Reviewing Method and Eligibility

Authors ran a peer review of the resulting articles based on their title and/or their abstract. Every article likely to be included in the systematic review was full-text analyzed. The following inclusion and exclusion criteria were applied.

### 2.4. Inclusion Criteria

Case-control and cohort studies analyze the connection between motor skills and language acquirement in HR children.

Subjects are no more than 36 months old by the last data collection record.

HR subjects must have direct relatives with ASD.

LR and control subjects must not have direct relatives with ASD.

Articles evaluate psychomotor skills, as well as communicative skills, using validated assessment tools.

### 2.5. Exclusion Criteria

Subjects who have any other motor or communicative disorder different from ASD.

Studies with no LR as a control group.

Non-scientific or opinion articles.

Not full-text articles.

The assessed outcome measures were: the age of the subjects at the time the study was concluded (≤36 months), the risk of having ASD (HR or LR) based on the existence of direct siblings with ASD, the evaluated psychomotor skills, the evaluated communicative skills, follow-up time, the assessment tools applied, methodological quality, the risk of bias, and results and conclusions.

### 2.6. Assessment of Methodological Quality and Risk of Bias

This systematic review was developed according to the PRISMA statement for reporting systematic reviews [25].

The methodological quality of each analyzed piece of research was assessed using the Newcastle–Ottawa Scale (NOS) [26], which determines the methodological quality based on the content, design, and usability of the analyzed literature. The NOS comprises eight items, split into three different dimensions (selection, comparability, and exposure). Each item grants a maximum of one star, except for ‘comparability’, which grants up to two stars, making a total of nine stars [27].

The risk of bias was assessed using the Cochrane risk-of-bias tool for randomized trials (RoB 2), which checks random sequence generation, allocation concealments, blinding of participants and personnel, blinding of outcome assessors, incomplete outcome data, selective reporting, and other bias. Each item can be assessed as “high risk of bias”, “low risk of bias” or “some concerns” [28].

## 3. Results

### 3.1. Study Selection

**Figure 1:**
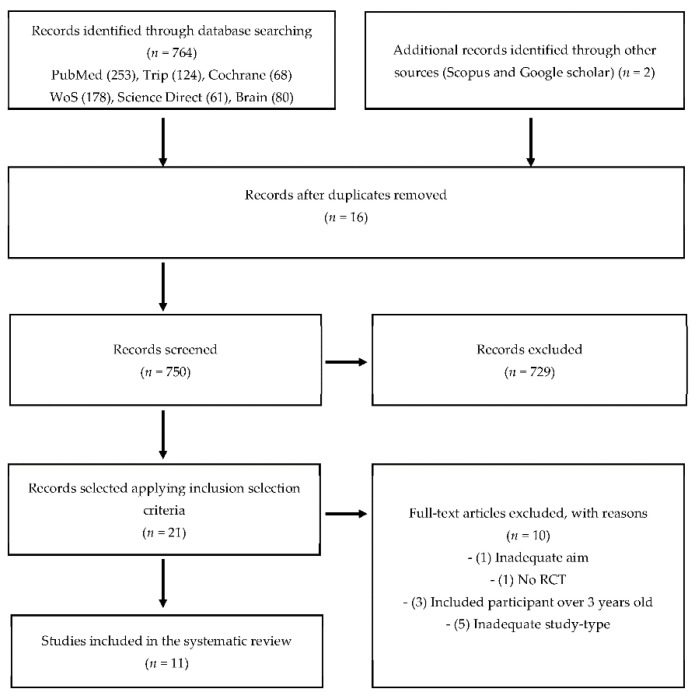
Flowchart. RTC: Randomized controlled trial.

Database searches resulted in 764 records, and two additional ones were identified through other sources (Scopus and Google Scholar) (Figure 1). Following the removal of duplicates, 16 articles were excluded. Thus, 750 articles were selected. Lastly, 729 articles were excluded based on the eligibility criteria previously defined. Twenty-one manuscripts were selected elected applying inclusion selection criteria. Finally, 11 articles were included [29,30,31,32,33,34,35,36,37,38,39], after excluding 10 for a variety of reasons: the intervention included an inadequate aim (*n* = 1), there was no control group (*n* = 1), or subjects were out of the age range (*n* = 3), and inadequate study-type (*n* = 5).

### 3.2. Study Characteristics

Every study [29,30,31,32,33,34,35,36,37,38,39] was an observational analytical cohort study. Besides the articles that carried out a global psychomotor analysis [30,33,34,35,36,37,38], two of them performed an analysis of gross and/or fine motor skills [29,37], one of them performed an analysis of fine motor skills and visual perception [31], another one analyzed gait milestones [39], and the last one looked at joint attention [32]. Joint attention is the shared visual attention between an adult and a child to an object pointed at by the adult.

Regarding language, four articles made a global analysis of communicative skills [29,30,33,36,37], and six carried out a specific analysis of expressive language [31,32,33,35,36,39]. The characteristics of these studies have been summarized in Table 1.

Every article implemented a 6- to 30-month-long monitoring period, previously distinguishing between subjects at an HR and LR of ASD. On top of that, five articles developed further classifications based on the ASD diagnosis tools results [31,35,37,38,39]. Seven articles assessed the communicative skills using both specific evaluation tools and ASD diagnosis tools [30,33,34,35,37,38,39].

### 3.3. Qualitative Analysis

In order to present the results and to develop a proper qualitative analysis, each outcome measure included in the selected literature has been connected to language development.

#### 3.3.1. Psychomotor Development and Language Development

Five articles made a general analysis of psychomotor skills and their relation with communicative skills and the language ability of the subjects [33,34,35,36,37,38]. All of them focused on intervention on the subject’s psychomotor development and the capacity to make strong predictions between language skills disorders and ASD diagnosis. Iverson et al., (2007) [34] presented conclusive results, associating fine motor skills at 6 months old with the prediction of ASD symptomatology at 36 months old. Iverson et al. (2007) [34] also proved the presence of both receptive and/or expressive vocabulary deficits in 9 out of 14 HR 18-month-old subjects. Besides this, two subjects that presented gait milestones deficits, and joint attention and first words disorders during the intervention subsequently had a positive ASD diagnosis. Landa et al. (2006) [35] demonstrated the worst motor and communicative skills results in the HR group with a later positive diagnosis (HRD), and a slower psychomotor development between 12 and 24 months old. LeBarton et al. (2019) [36] described motor skills at 6 months old as a reliable predictive outcome measure for ASD at 24–36 months old. Based on this data, expressive communication skills could also be predicted at 30–36 months. Leonard et al. (2015) [38] analyzed psychomotor development. Results showed that expressive deficits in ASD subjects could be predicted based only on both fine and gross motor skills.

#### 3.3.2. Gross Motor Skills, Fine Motor Skills, and Language Development

Bhat et al. (2012) [29] compared early gross motor skills between HR and LR groups, as well as their relation with language disorders. Results demonstrated that 78% of HR subjects scored lower in gross motor skills tests at 3 months old, in contrast with 33% of LR subjects. At 6 months old, low scores in gross motor skills tests were seen in 50% of HR subjects in contrast with 8.3% of LR subjects. Half of the HR subjects presented both motor and communicative deficits. LeBarton et al. (2013) [36] confirmed a delay in fine motor skills development, between 12 and 24 months old, in 86% of HR subjects later diagnosed with ASD. A reliable prediction of expressive language at 36 months old based on fine motor skills at 12–18 months old was also confirmed in both LR and HR groups.

#### 3.3.3. Fine Motor Skills, Visual Perception and Language Development

Choi et al. (2018) [31] retroactively classified HR subjects depending on whether they had a positive or negative diagnosis for ASD after the intervention (hereon HRD and HRND respectively). Results showed worst fine motor skills scores in HRD subjects in comparison with HRND and LR subjects at 12 months old. Thus, the results regarding fine motor skills prove to be a reliable predictor to detect expressive language deficits. Correlative differences related with visual perception were not found in any of the groups.

#### 3.3.4. Motor Imitation, Joint Attention and Language Development

Bruyneel et al. (2019) [30] simultaneously analyzed motor skills, communicative skills and joint attention. It was proved that joint attention has a relevant role for LR and HR groups, with HR subjects being more vulnerable to having language disorders if they show both motor and joint attention deficits at the same time. Edmunds et al. (2017) [32] evaluated motor imitation, expressive communication skills, and joint attention. Results showed that motor imitation skills at 12 months old predict expressive vocabulary at 18 months old.

#### 3.3.5. Gait and Language Development

West et al. (2019) [39] carried out a specific assessment of motor skills in two different periods: transition towards gait achievement and gait achievement. The results of both periods were later related with their language prediction capacity. Post-intervention classifications were made, dividing HR subjects in HRD, HRND, and HR subjects with language disorders (HRLD). Significantly lower scores were achieved by HR and HRLD in every communicative outcome measure when compared to LR and HRND groups.

#### 3.3.6. Assessment of Methodological Quality and Risk of Bias

A summary of authors’ judgements about the methodological quality of the articles was performed. The Newcastle–Ottawa Scale (NOS) for assessing the quality of nonrandomized studies in meta-analyses was applied to each of the articles. Two articles scored 9/9 points [31,35], six scored 8/9 [29,30,32,33,34,38], and three scored 7/9 [36,37,39].

A revised tool to assess risk of bias in randomized trials (RoB 2) was applied to each article. Six articles were found to have a “low risk of bias” [29,30,32,33,34,36], four articles were found to have “some concerns” [31,35,38,39], and one article was found to have a “high risk of bias” [36].

## 4. Discussion

The findings of this systematic review demonstrate that a reliable prediction of language disorders and/or ASD can be made based on the early psychomotor development of HR children. Diverse psychomotor skills have been assessed. Gross and fine motor skills have been the most specifically measured parameters [29,31,36]. Even in those studies whose purpose was not their precise assessment, gross and fine motor skills have shown significant outcomes for ASD prediction [33,35,38]. In this manner, Bath et al. (2012) [29] proved that 78% of HR subjects achieved significantly worse scores in gross motor skills than their LR peers. In addition, a direct relationship between the development of motor skills and the prediction of communicative skills of the subjects is determined. This data is consistent with Leonard et al. (2015) [38], who suggest that expressive language at 36 months old is predicted by gross motor skills data at 7 months old.

As for the study of fine motor skills, more reliable data is produced in various studies owing to a greater statistical significance. Some studies, like LeBarton et al. (2013) [36], exposed a developmental delay in the acquirement of fine motor skills in 86% of HR subjects at 12–24 months old. Furthermore, a significant prediction of language development at 36 months old was made, based on fine motor skills acquirement data. In relation with these fine motor skills conclusions, some similarities were found in Choi et al. (2018) [31]. Their research proves a significantly worst development of fine motor skills at 12 months old in the HRD group. Finally, Leonard et al. (2015) [38] demonstrated that language disorders at 36 months old can be predicted based on fine motor skills data. The data in these studies establish a direct link between deficits in early fine motor skills and the prediction of language disorders, especially in HR subjects for ASD.

Regarding joint attention, both Bruyneel et al. (2019) [30] and Edmunds et al. (2017) [32] affirmed that when joint attention and gross motor skills (Bruyneel et al., 2019) [30] or motor imitation (Edmunds et al., 2017) [32] deficits are seen simultaneously in HR children, subjects are more likely to develop language disorders. These conclusions strengthen the presumption of joint attention as a pre-linguistic process, which is coherent with the fact that communicative development in HR children is altered.

Even though the article by Iverson et al. (2007) [34] was not designed for the specific analysis of gait development, but for the study of the development of psychomotor skills, significant outcomes were found. According to Iverson et al. (2007) [34], 100% of HRD subjects exhibited delays in gait milestones achievement. By contrast, those gait milestones were assessed in a specific way by West et al. (2019) [39]. When assessments concurred with the main gait milestones, more difficulties for producing and comprehending words were found in HRD subjects, compared with their HR and LR peers. Data presented on both articles suggest that gait milestones are sensitive moments for the prediction of communicative disorders associated to ASD. In this manner, the development of motor skills in this specific period of time could also be relevant for the prediction and an early diagnosis of ASD. This is so, in the first place, because HRD subjects show significantly worst gait skills and delays in their consecution (Iverson et al., 2007) and, in the second place, because of the connection between this period and language development (West et al., 2019) [39].

In relation to the prediction of language in HR subjects, similar outcomes were obtained in each study. More specifically, prediction of expressive language tends to be more reliable compared with receptive language prediction. This is due to the fact that expressive language can be significantly predicted based on fine motor skills [31,36], motor imitation, and joint attention (Edmunds et al., 2017) [32], first words production (Iverson et al., 2007), gross motor and general motor skills [29,36,38]. These predictions are mostly made for subjects 36 months old.

As for the age ranges analyzed in the literature, the period between 12 and 36 months old is considered to be the most decisive for the prediction of language disorders related to ASD, based on early psychomotor skills. Even though psychomotor skills assessments were made before age 12 months [29,30,33,36], most of the literature evaluated those skills after age 12 months [31,32,34,35,36,38,39].

It is important to emphasize that, in every article analyzed, outcome measures and ASD assessment tools can be combined to diagnose both ASD and related language disorders.

With reference to the methodological quality, and after the literature analysis, “representativeness of cases” was the most common item for which some articles did not obtain positive scores in the NOS scale. This is so because the selection of HR population for the studies is done in close collaboration with ASD associations and ASD support groups. Thus, HR and LR subjects cannot be selected from the same source. Acceptable results were revealed in the rest of items after the assessment and analysis with the NOS scale. Concerning the analysis of the risk of bias, “high risk of bias” is proved for one article [37]. This is caused by the second intervention of the study, in which only the HR group was admitted, excluding the LR group previously included in the first intervention.

During the search for literature, exclusively articles in Spanish and English were selected. Because of this reason, the articles’ language is identified as a limitation of the systematic review.

## 5. Conclusions

After evaluating every psychomotor outcome measure included in the literature, fine motor skills have been identified as the most analyzed and reliable outcome measure in order to predict expressive language disorders linked to ASD and its diagnosis in HR subjects. Likewise, authors find it essential to emphasize the relevance of joint attention in the prediction of language disorders linked to ASD, especially because of the connection between this ability, language, and socio-emotional development. After a deep analysis, 12–24 months old has been proved as the most reliable age range to properly evaluate psychomotor skills in order to predict ASD and related language disorders. Pointedly, it is from age 24 months when the best and most reliable outcomes are found. In this process, the importance of language development and gait milestones is also highlighted. In relation to the prediction of language disorders and ASD, data at 36 months old is the most reliable, in comparison with outcomes before that age.

## Figures and Tables

**Table 1:** Summary of studies.

Study	Intervention Type	Sample Size Age (Months) Sex	Outcome Measures Age of Assessment (Months)	Tools Assessments	Results	NOS
Btah et al. (2012) [29]	Longitudinal observational analytic cohort study	HR (*n* = 24) M (*n* = 12) F (*n* = 12) LR (*n* = 24) M (*n* = 9) F (*n* = 15) (3–18) M (*n* = 21) F (*n* = 27)	Gross motor skills: AIMS (3/6) Motor and communicative skills: MSEL (18)	AIMS (*n* = 2) MSEL (*n* = 1)	67–73% of HR subjects that presented early motor skills disorders subsequently presented delays in communicative skills.	NOS: 8/9
Bruyneel et al. (2019) [30]	Longitudinal observational analytic cohort study	HR (*n* = 32) LR (*n* = 31) (10–36) M (*n* = 32) F (*n* = 31)	Motor and communicative skills: MSEL (10/14/36) ASD diagnosis: ADOS-2 (14)	MSEL (*n* = 3) ADOS-2 (*n* = 1)	Fine and gross motor skills at 10 months old had a direct impact on expressive language skills (HR and LR) at 36 months old. Poor motor skills implied a trigger effect on both joint attention and language development in HR subjects. Reliable predictions of language disorders could be made based on early motor skills of HR subjects.	NOS: 8/9
Choi et al. (2018) [31]	Longitudinal observational analytic cohort study	HRND (*n* = 71) M (*n* = 33) F (*n* = 38) HRD (*n* = 30) M (*n* = 21) F (*n* = 9) LR (*n* = 69) M (*n* = 38) F (*n* = 31) (6–36) M (*n* = 92) F (*n* = 78)	Motor and communicative skills: MSEL (6/12/18/24/36) Fine motor skills (6/12/18/24) Expressive language (36) Visual perception (6)	MSEL (*n* = 5) ADOS	Fine motor skills development between 6 and 24 months old was significantly slower in HRD than in HRND and LR subjects. Fine motor skills development allowed to predict expressive communicative skills at 36 months old.	NOS: 9/9
Edmunds et al. (2017) [32]	Longitudinal observational analytic cohort study	HR (*n* = 50) M (*n* = 29) F (*n* = 21) LR (*n* = 34) M (*n* = 16) F (*n* = 18) (12–18) M (*n* = 45) F (*n* = 39)	Motor imitation: STAT (12) Joint attention: ESCS (12/15) Expressive language: CDI (12/15/18)	STAT (*n* = 1) ESCS (*n* = 2) CDI (*n* = 3)	Motor imitation was directly related with the prediction of expressive vocabulary and joint attention in both HR and LR. ASD developed expressive vocabulary acquisition disorders.	NOS: 8/9
Iverson et al. (2019) [33]	Longitudinal observational analytic cohort study	HR (*n* = 437) M (*n* = 256) F (*n* = 181) LR (*n* = 188) M (*n* = 107) F (*n* = 81) (6–36) M (*n* = 363) F (*n* = 262)	Motor and communicative skills: MSEL (6) ASD diagnosis: ADOS (36)	MSEL (*n* = 1) ADOS (*n* = 1)	Lower marks in gross and fine motor skills were shown by HR subjects; fine motor skills data being more reliable. Significant differences between HRD and HRND/ND groups were found. Only fine motor skills data at 6 months old was able to predict ASD severity at 36 months old based on ADOS.	NOS: 8/9
Iverson et al. (2007) [34]	Longitudinal observational analytic cohort study	HR (*n* = 21) M (*n* = 6) F (*n* = 15) LR (*n* = 18) M (*n* = 8) F (*n* = 10) (5–18) M (*n* = 14) F (*n* = 25)	Expressive language: CDI (8 a 18) Motor skills and ASD diagnosis: PDDST-II (18)	CDI (*n* = 11) PDDST-II (*n* = 1)	The HR group presented a significant delay on the achievement of developmental milestones (independent stable sitting, posture, language development, rhythmic movements and babbling). Language reception and execution delays on 64.2% of subjects at 18 months old.	NOS: 8/9
Landa et al. (2006) [35]	Longitudinal observational analytic cohort study	HR (*n* = 60) M (*n* = 35) F (*n* = 25) LR (*n* = 27) M (*n* = 17) F (*n* = 10) HRD (*n* = 24) LD (*n* = 11) ND (*n* = 52) (6–24) M (*n* = 52) F (*n* = 35)	Motor and communicative skills: MSEL (6/14/24) Expressive language: CDI (14/24) ASD diagnosis: PDS-III/IV y ADOS (24)	MSEL (*n* = 3) CDI (*n* = 2) PDS (III-IV) (*n* = 1) ADOS (*n* = 1)	HR and LR are later classified depending on whether they present positive ASD diagnosis, negative ASD diagnosis, or language disorders. No significant differences at 6 months old. Worst results in every assessed item (except for visual perception) in HRD group at 14 months old. Worst results in every assessed item in HRD group at 24 months old. HRD follow-up was significantly worst, especially between 12 and 24 months old.	NOS: 9/9
LeBarton et al. (2013) [36]	Longitudinal observational analytic cohort study	Intervention 1: HR (*n* = 34) M (*n* = 18) F (*n* = 16) LR (*n* = 25) M (*n* = 10) F (*n* = 15) Intervention 2: HR (*n* = 34) M (*n* = 18) F (*n* = 16) (12–36) M (*n* = 28) F (*n* = 31)	Fine motor skills: IOM (12/18) Motor and communicative skills: MSEL (24/36) Expressive language: CDI (36)	IOM (*n* = 2) MSEL (*n* = 3) CDI (*n* = 1)	Intervention 1: 86% of HRD subjects developed fine motor skills delays between 12 and 24 months old. Intervention 2: expressive language development at 36 months old was significantly predictable by IOM at (12/18 months) and fine motor skills MSEL scale (24 months).	NOS: 7/9
LeBarton et al. (2019) [37]	Longitudinal observational analytic cohort study	HRND (*n* = 69) HRD (*n* = 20) LR (*n* = 51) (6–36) M (*n* = 79) F (*n* = 61)	Motor skills: PDMS-2 (6) Motor and communicative skills: MSEL (6/24/30/36) ASD diagnosis: ADOS-2 (24/30/36)	PDMS-2 (*n* = 1) MSEL (*n* = 4) ADOS-2 (*n* = 3)	Intervention 1: motor skills at 6 months old predicted ASD diagnosis at 24/36 months old. Intervention 2: MSEL as dependent variable. Grabbing and stationary scales predicted expressive language at 30 and 30/36 months old respectively.	NOS: 7/9
Leonard et al. (2015) [38]	Longitudinal observational analytic cohort study	HRND (*n* = 36) M (*n* = 10); F (*n* = 26) HRD (*n* = 17) M (*n* = 11) F (*n* = 6) LR (*n* = 48) M (*n* = 17) F (*n* = 31) (7–36) M (*n* = 38) F (*n* = 63)	Motor and communicative skills: MSEL (7/14/24/36) Communicative capacities: VASB-II (7/14/24/36). ASD diagnosis: ADOS-G, ADI-R and ICD-10 (36)	MSEL (*n* = 4) VABS-II (*n* = 4) ADOS-G (*n* = 1) ADI-R (*n* = 1) ICD-10 (*n* = 1)	A link was detected between deficits in gross and fine motor skills, and later deficits on expressive language. Fine motor skills data was less significant.	NOS: 8/9
West et al. (2019) [39]	Longitudinal observational analytic cohort study	HRD M (*n* = 10) F (*n* = 5) HRND M (*n* = 23) F (*n* = 27) HRLD M (*n* = 15) F (*n* = 11) LR (*n* = 25) M (*n* = 10) F (*n* = 15) (18–36) M (*n* = 58) F (*n* = 58)	Gait development: Video records Motor and communicative skills: MSEL (18/24/36) Expressive language: CDI (18/24/36) ASD diagnosis: ADOS (36)	MSEL (*n* = 3) CDI (*n* = 3) ADOS (*n* = 1)	Only HRD subjects did not acquire language skills after the achievement of gait milestones.	NOS: 7/9

HR: high risk; LR: low risk; M: male; F: female; HRND: high risk not diagnosed; HRD: high risk diagnosed; LD: language disorder; ND: not diagnosed; n: number; ASD: Autism spectrum disorder; NOS: Newcastle–Ottawa quality assessment scale; MSEL: Mullen scales of early learning; AIMS: Alberta infant motor scale; ADOS: autism diagnostic observation schedule; STAT: screening tool for autism in toddlers; ESCS: early social communication scales; CDI: MacArthur–Bates communicative development inventory: words and gestures; PDDST-II: pervasive developmental disorder screening test; PDS (III-IV): preschool language scale; VASB-II: Vineland adaptive behavior scales-II; IOM: infant oral and manual motor interview; PDMS-2: Peabody developmental motor scales—2; ADI-R: autism diagnostic interview—revised; ICD-10: consensus ICD-10.

## Data Availability

Not applicable.
